# The spatiotemporal movement of patients in and out of a psychiatric hospital: an observational GPS study

**DOI:** 10.1186/s12888-021-03147-9

**Published:** 2021-03-24

**Authors:** Andrew T. Gloster, Andrea H. Meyer, Jens Klotsche, Jeanette Villanueva, Victoria J. Block, Charles Benoy, Marcia T. B. Rinner, Marc Walter, Undine E. Lang, Maria Karekla

**Affiliations:** 1 University of Basel, Department of Psychology, Division of Clinical Psychology & Intervention Science, Missionsstrasse 62A, CH-4055 Basel, Switzerland; 2 University of Basel, Department of Psychology, Division of Clinical Psychology & Epidemiology, Basel, Switzerland; 3 German Rheumatism Research Center Berlin, Epidemiology unit and Charité Universitaetsmedizin Berlin, Institute for Social Medicine, Epidemiology and Health Economics, Berlin, Germany; 4 University Psychiatric Clinics (UPK), University of Basel, Basel, Switzerland; 5 University of Cyprus, Department of Psychology, Nicosia, Cyprus

**Keywords:** Spatiotemporal movement, Wellbeing, Global positioning system (GPS), Transdiagnostic

## Abstract

**Background:**

Movement is a basic component of health. Little is known about the spatiotemporal movement of patients with mental disorders. The aim of this study was to determine how spatiotemporal movement of patients related to their symptoms and wellbeing.

**Method:**

A total of 106 patients (inpatients (*n* = 69) and outpatients (*n* = 37)) treated for a wide range of mental disorders (transdiagnostic sample) carried a GPS-enabled smartphone for one week at the beginning of treatment. Algorithms were applied to establish spatiotemporal clusters and subsequently related to known characteristics of these groups (i.e., at the hospital, at home). Symptomatology, Wellbeing, and Psychological flexibility were also assessed.

**Results:**

Spatiotemporal patterns of inpatients and outpatients showed differences consistent with predictions (e.g., outpatients showed higher active areas). These patterns were largely unassociated with symptoms (except for agoraphobic symptoms). Greater movement and variety of movement were more predictive of wellbeing, however, in both inpatients and outpatients.

**Conclusion:**

Measuring spatiotemporal patterns is feasible, predictive of wellbeing, and may be a marker of patient functioning. Ethical issues of collecting GPS data are discussed.

**Supplementary Information:**

The online version contains supplementary material available at 10.1186/s12888-021-03147-9.

## Introduction

Life entails movement. Some organisms respond to stimuli with changes viewable only in a microscope. Others appear immobile, but bend towards sunlight for energy. Yet others run 42.2 km without being hunted. While the functions of movement differ across organisms and time, the ubiquity of movement suggests that it is a fundamental contributor to an organism’s fitness and each organism must gage how and when to exert the necessary effort to achieve their goals [1].

Within humans, physical activity is believed to be beneficial for people’s mental and physical health and low levels of movement may be associated with ill-health [2]. Indeed, people diagnosed with mental disorders may limit their mobility for reasons related to the disorder. For example, the lack of energy often experienced during depression may encumber one’s ability to move and engage in activities [3, 4]. Other diagnoses such as anxiety disorders suggest limited mobility. Examples include avoidance of leaving safe places (e.g., agoraphobia [5], panic or phobias). People suffering from other mental disorders may limit their mobility due to pain or other bodily sensations (i.e., somatic symptom disorder) [6]. Yet others may not limit the quantity of their movement, but rather the variability of the places they visit. This may be due to lack of energy, or in the desire to avoid having symptoms detected by others such as in obsessive-compulsive disorder, social anxiety, or psychosis. To date, it remains unclear how spontaneous movement during the navigation of daily life is impacted in patients presenting for treatment.

It is also unclear how restricted or stereotypical movement patterns, if they exist, are associated with patients’ wellbeing. That is, the degree to which one experiences positive feelings (emotional wellbeing), and functions well in one’s own life (psychological wellbeing) and the community (social wellbeing) [7]. Although the relationship between physical movement and wellbeing is unknown in patients, studies on exercise in the general population suggest that higher levels of wellbeing could be associated with movement patterns. Conversely, given that wellbeing and symptomatology are not simply opposites, there may be no relationship between wellbeing and movement.

How people respond to their symptoms mitigates their impact [8]. Therefore, when exploring the importance of patients’ movement, it is also important to examine how movement patterns relate to psychological responses that contribute to mental health. One such variable is psychological flexibility, or the ability to be psychologically present while engaging in the things one deeply cares about *despite* uncomfortable thoughts or emotions [9, 10]. It is probable, though untested, that someone high in psychological flexibility is more likely to engage in the varied activities that serve their chosen values and this might be reflected in their movement patterns.

Given that very little is known about the broader swath of spontaneous spatiotemporal movements of patients with mental disorders – and their association with wellbeing and psychological flexibility – it is important to understand whether such a basic contributor of health as physical movement patterns is compromised in patients with mental disorders. To our knowledge, information on the natural spatiotemporal movements of participants (i.e., using the Global Positioning System [GPS]) diagnosed with a mental disorder have been limited to studies that included either non-clinical participants [11], or only a handful of patients [12], and no studies exist that examine this information across various diagnoses and treatment modalities (i.e., inpatient and outpatient treatment).

Thus, the aim of this study is to document patients’ spatiotemporal movement (e.g., how much participants move and in which way) during the first week of treatment. To add to the generalizability of the findings, transdiagnostic patients were examined in two different treatment settings, namely inpatients and outpatients. As a proof of method, we hypothesized that outpatients would have greater indices of spatiotemporal activity than inpatients (Hypothesis 1). Further, we hypothesized that independent of treatment modality, lower levels of symptomatology (Hypothesis 2) and higher levels of wellbeing and psychological flexibility (Hypothesis 3) would be associated with spatiotemporal patterns.

## Methods

### Design

GPS data collection occurred within a seven-day-Event Sampling Methodology (ESM) phase within a longitudinal, controlled clinical effectiveness trial [13].

### Participants

Participants (*n* = 106; Inpatients: *n* = 69; outpatients: *n* = 37) were recruited from two specialized units within a psychiatric hospital with an open-door policy in Switzerland, from ongoing intake procedures. Inclusion criteria were: ≥ 18 years, ability to speak German. Exclusion criteria were: acute suicidal intent, acute substance dependency, active mania, and inability to read or complete assessments. Otherwise, all diagnoses were eligible (e.g., Affective Disorders, Anxiety Disorders, Somatoform Disorders, Mood Disorders, Anxiety-stress related Disorders, Somatic Disorders, Obsessive-Compulsive Disorder, Impulse Control Disorders, and Personality Disorders). Participants completed informed consent and were explicitly informed about and consented to GPS data collection.

### Procedure

Participants carried a study-issued smartphone (i.e., not their personal phone), which was set to automatically collect GPS data as soon as the smartphone was turned on to avoid data loss if the phone was shut down during the assessment week. Participants were instructed to carry the phone with them during the study week. Participants explicitly gave permission to activate GPS on the study phones. They were informed that the studyphones (with disabled wi-fi and no SIM-card) would not be trackable and location data would be saved locally on the phone. Patients were free to leave the ward any time. Further, all patients were highly encouraged to partake in individual engagement exercises, which involved engaging in activities that kept them in contact with the important aspects of their life. Most patients went home weekends and some went home nights.

### Assessments

#### GPS

GPS data was automatically logged every five seconds as a balance between high frequency data collection and battery life. GPS data were subsequently converted for analysis (latitude, longitude, date, time of day). GPS data for one patient on one particular day were only included if ≥ 1000 signals were available, which corresponded to GPS data for at least 1.4 h per day. The theoretically maximum number of GPS points was 17,280 for 24 h with recordings every 5 s. Subsequently, the ST-SBCAN algorithm – a state-of-the-art density-based clustering algorithm [14] – was applied individually for each patient and day and the obtained spatiotemporal clusters were merged with the GPS coordinates of the hospital (in the case of inpatient) and home (in case of outpatient) location of each patient. Coordinates of the hospital and home were defined in decimal degrees, and all destinations with centroid coordinates within a radius of 200 m of the hospital or home coordinates were given the respective label. All data points included in any estimated cluster (see below) were labeled by “not in-transit”. Finally, all data points that were not included in any of the estimated clusters were grouped in one cluster labelled “in-transit” to indicate that patient were moving between two clusters.

#### Questionnaires

Symptoms were assessed using the Brief Symptom Checklist (BSCL) [15], a 53-item self-report inventory measuring levels of psychopathology on a scale from 0 (not at all) to 4 (extremely). The nine subscales show sufficient to good internal consistency (Cronbach’s α = .75 to .90). Wellbeing was assessed using the Mental Health Continuum – Short Form (MHC-SF) [7], a self-report inventory consisting of 14 items that show high internal consistency (Cronbach’s *α* > .80). Each item assesses how often a statement was true during the past month, ranging from 0 (never) to 5 (almost every day). Psychological flexibility was measured using the Psyflex scale ([16]; Gloster AT, Block VJ, Klotsche J, Villanueva J, Rinner MTB, Benoy C, et al: Psy-Flex: A Contextually Sensitive Measure of Psychological Flexibility, In review) which measures core skills of psychological flexibility on a scale from 1 (very rarely) to 5 (very often). The Psyflex shows very high internal consistency (Raykov’s *r* = 0.91) and produces a single score with higher scores indicating better psychological flexibility qualities.

### Data processing

**Fig. 1:**
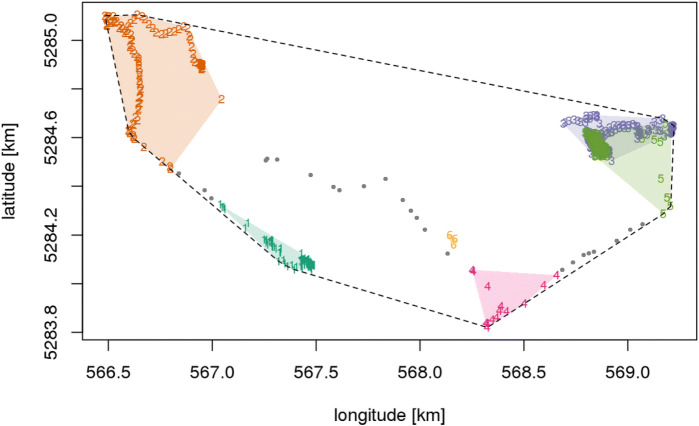
Example of Spatiotemporal Movement of one person. Spatiotemporal activity pattern of a selected patient during one day. The ST-DBSCAN algorithm estimated six clusters in this case. Numbers denote GPS signals of a specific cluster in temporal order of recording (1 = first cluster, 6 = last cluster recorded throughout the day). Color-shaded areas refer to the hulls around individual clusters. The broken line denotes the hull boundary around all GPS signals recorded during the entire day. Grey dots denote GPS signals allocated to “in transit” destinations. 1=turquoise, 2=brown, 3=violet, 4=red (carmine), 5=green, 6=yellow

Patients with more than 1000 GPS coordinates per day were included into the analysis. The ST-DBSCAN algorithm was used to identify unique destinations for each patient within a day [17]. The ST-DBSCAN algorithm contains three parameters that had to be assigned before estimating unique destinations: spatial distance, temporal distance, and number of points needed to form a cluster (see Additional file 2 for details). Thus, a destination was comprised of at least 10 data points which were all within a Euclidean distance of 200 m with a temporal proximity of 20 min [12]. The following measures were calculated from the GPS data points for each patient and day after completing the ST-DBSCAN algorithm (see Fig. 1 for an example).

- The *individual hull area*, i.e. the area (in km^2^) under the minimum convex polygon that includes all GPS data points of a unique cluster. It is the shortest possible line that surrounds all GPS points of a unique cluster with an outward curvature. The resulting area does not have any indentation.
- The *total individual hull* area (in km^2^) is the sum over all individual hull areas across one day.
- The *total hull area* (total activity area; in km^2^) is the area under the minimum convex polygon (see explanation above for the individual hull area) that includes all GPS data points across one day (disregarding unique clusters). The total hull area is always greater or equal to the sum over all individual hull areas.
- The *distance travelled within destinations* is the cumulative distance (in km) among all GPS data points within a cluster.
- The *distance travelled across the entire day* is the cumulative distance (in km) across all GPS data points, disregarding unique clusters.
- The *time spent within a cluster* (in minutes).
- The variability measures *entropy* and *normalized entropy* [11]. It is a measure to describe the variability of the time within a destination for a patient at a given day. For more details, see Saeb et al. [11]. A high entropy means that the patient has spent his/her time more evenly distributed over the clusters per day. In contrast, low entropy values mean that the time spent within a cluster varies within a patient at a given day.
- The *location variance* is a summary measure of the statistical variances of latitude and longitude [11] to determine the variability in patient’s destination. “In transit” destinations were not included in the calculation of location variance. Location variance is estimated by the logarithm of the sum of variances of latitude and longitude per patient and day.

The ST-DBSCAN function and all other functions necessary to calculate the measures mentioned above were taken from the statistical software R [18].

### Statistics

Descriptive statistics included mean and standard deviation and median and interquartile range (25th percentile and 75th percentile). Due to the hierarchical nature of the data (clusters within patients per day), we used weighted statistics, i.e., patients with more clusters gave more weight to the calculated statistics. Results are reported for the entire sample, inpatients and outpatients, as well as for different types of destinations. The home address was not known for some patients (*n* = 17, covering 52 days) hence the cluster status could not be unequivocally allocated. *P*-values are based on *Mann-Whitney test* (comparison between in- and outpatients) and Spearman rank correlation coefficients (associations with continuous variables), whereby spatiotemporal data were first averaged across all clusters and days to obtain one value per patient.

## Results

### Patients and days

Data from 106 patients were available for this study. The mean age was 34.8 years (SD =11.4, median = 32.3) and 56 (52.8%) of the patients were males (Table 1). The sample included 69 inpatients and 37 outpatients. The total number of days analyzed was 448, with an average of 3.7 (SD = 2.1, median = 3) days per patient. The mean number of GPS signals recorded was 5.4 (SD = 2.4, median = 5.2) per minute resulting in a mean number of 6′013 GPS signals per patient and day. The mean percentage of time covered by GPS recordings over 24 h was 62% (SD = 34.2%, median = 58%, minimum = 8%, maximum = 100%) with inpatients covering on average a lower percentage of time by GPS recordings (mean = 46.1%, SD = 25.7, median = 39.4%) than outpatients (mean = 81.3%, SD = 33.2, median = 84.7%).

**Table 1:** Patient characteristics (*n* = 84)

	n/mean	%
Sex		
Female	38	46.2
Male	46	54.8
Age (years)		
Mean	34.2	
SD	11.1	
Min – Max	17.7–64.1	
Marital status		
Married/ partnership	26	31
Single	38	45
Divorced/ separated	5	6
No information	15	18
Adults in the household		
None	2	2
One	23	27
Two	20	24
More than two	22	26
No information	17	20

### Number of clusters

A total of 3769 clusters were identified, using the parameter settings from the ST-DBSCAN algorithm as described in the methods section. Of these, 3482 (92%) referred to “not in-transit” and 287 (8%) to “in transit” destinations (i.e., participants were moving too fast to be attributed to any other cluster and therefore were in transit). The proportion of “in transit” destinations was somewhat higher for outpatients (152 [8.8%] of 1728) compared to inpatients (135 [6.6%] of 2041). The mean number of destinations for a patient within a day was 8.4 and hardly varied between in- and outpatients (Table 2). The average number of destinations per day was higher in outpatients than inpatients for home locations (1.7 versus 0.6), locations other than home or hospital (5.0 versus 2.9), and in transit (0.8 versus 0.5) but much lower for the destination “at the hospital” (0.1 versus 3.8).

**Table 2:** Number of destinations and number of destinations per day estimated by the parameter setting *eps1* = 200 m, *eps2* = 20 min and *minpts* = 10 of the ST-DBSCAN algorithm

	Inpatients	Outpatients	*p* value	All
	(*n* = 53)	(*n* = 31)	(inpatients versus outpatients)	(n = 84)
Total number of destinations	2041 (100.0%)	1728 (100.0%)		3769 (100.0%)
at home	139 (6.8%)	345 (20.0%)	< 0.001	484 (12.8%)
at hospital	932 (45.7%)	14 (0.8%)		946 (25.1%)
at other location	726 (35.6%)	996 (57.6%)		1722 (45.7%)
in transit	135 (6.6%)	152 (8.8%)		287 (7.6%)
location unclear^a^	109 (5.3%)	221 (12.8%)		330 (8.8%)
Average number of destinations per patient and day^b^	8.2 (4.5)	8.6 (6.0)		8.4 (5.2)
	8 (5–10)	8 (4–12)	0.935	8 (5–11)
at home^b^	0.6 (1.3)	1.7 (1.9)		1.1 (1.7)
	0 (0–0)	1 (0–3)	< 0.001	0 (0–2)
at hospital^b^	3.8 (3.2)	0.1 (0.3)		2.1 (3.0)
	3 (1–6)	0 (0–0)	< 0.001	0 (0–4)
at other location^b^	2.9 (4.0)	5.0 (5.6)		3.8 (4.9)
	2 (0–4)	3 (0–8)	< 0.001	2 (0–6)
in transit^b^	0.5 (0.5)	0.8 (0.4)		0.6 (0.5)
	1 (0–1)	1 (1–1)	< 0.001	1 (0–1)
location unclear^a,b^	0.4 (1.8)	1.1 (3.0)		0.7 (2.4)
	0 (0–0)	0 (0–0)	0.002	0 (0–0)
Summed activity area (sum of all individual cluster areas) (km^2^)	15.1 (83.9)	106.4 (496.3)		55.8 (340.0)
	0.30 (0.04–2.36)	2.12 (0.25–10.14)	< 0.001	0.69 (0.06–3.72)
Percent time within a cluster with respect to the recorde total time				
at home^b^	7.3 (14.4)	29.6 (31.0)		15.5 (24.4)
	0.0 (0.0–6.6)	31.2 (0.0–57.8)	< 0.001	0.0 (0.0–30.4)
at Hospital^b^	45.9 (28.2)	0.6 (2.1)		29.2 (31.4)
	43.1 (22.1–71.2)	0.0 (0.0–0.0)	< 0.001	16.5 (0.0–56.5)
at other location^b^	22.3 (20.0)	22.1 (23.8)		22.2 (21.3)
	18.3 (3.7–33.6)	13.0 (0.0–43.5)	0.586	17.6 (3.2–37.0)
in transit^b^	20.0 (18.6)	26.6 (16.9)		22.4 (18.2)
	16.1 (2.7–30.6)	25.7 (14.3–41.3)	0.058	20.2 (5.1–32.6)
location unclear^a,b^	4.6 (16.1)	21.1 (37.0)		10.7 (26.9)
	0.0 (0.0–0.0)	0.0 (0.0–55.1)	0.084	0.0 (0.0–0.0)

^a^ Locations unclear since home address not known and cluster status therefore not unequivocally attributable

^b^ Statistics denote mean (SD) at first row, and median (25. – 75. percentile) at second row

Home destinations made up 13% of all destinations and were more frequent in outpatients (20%) than inpatients (6.8%). In inpatients, hospital destinations made up almost half (46%) of all destinations, in outpatients in contrast only 0.8%. Finally, 46% of all destinations concerned destinations other than “in transit”, “at home”, “at the hospital”, or “location unclear” and this percentage was clearly higher in outpatients (58%) than inpatients (36%).

A sensitivity analysis was performed by selecting only patients for whom more than 60% of all daily GPS signals were recorded. This was done in order to assess the influence of missing GPS recordings on the results. In total 233 (out of 448) days (1636 destinations) were excluded this way. We then generated an additional table (see Additional file 1), which corresponded to Table 2 of the manuscript but was based on the restricted dataset. The basic parameters such as the distribution of the frequency destinations within a day, the time spent within a destination or the percentage of time within a cluster in outpatients were all comparable between these two approaches (Additional file 1). The relative number of “hospital” destinations in inpatients dropped somewhat in favor of the other destinations.

### Properties of destinations

The distributions of properties of destinations were highly skewed. As expected, the total activity area was larger than the sum across all individual cluster areas (median, 1.08 km^2^ versus 0.69 km^2^, Table 2 and Additional file 2). Outpatients showed a considerably higher summed individual activity area (median, 2.12 km^2^) compared to inpatients (median, 0.30 km^2^). Outpatients spent 31% (median) of the day within the home destination, whereas inpatients were in median 0% of the time at home. In contrast, inpatients spent almost half of the day in the hospital (median 43.1%) and outpatients not (median 0%). In addition, outpatients were significantly more time of the day in transit (median 25.7% versus 16.1%). See Additional file 2 for additional properties of the destinations. In summary, all the results are in line with the expectation about the difference between inpatients and outpatients (i.e., higher mobility and larger activity area).

### Relation between spatiotemporal movement and symptomatology (hypothesis 2)

**Fig. 2:**
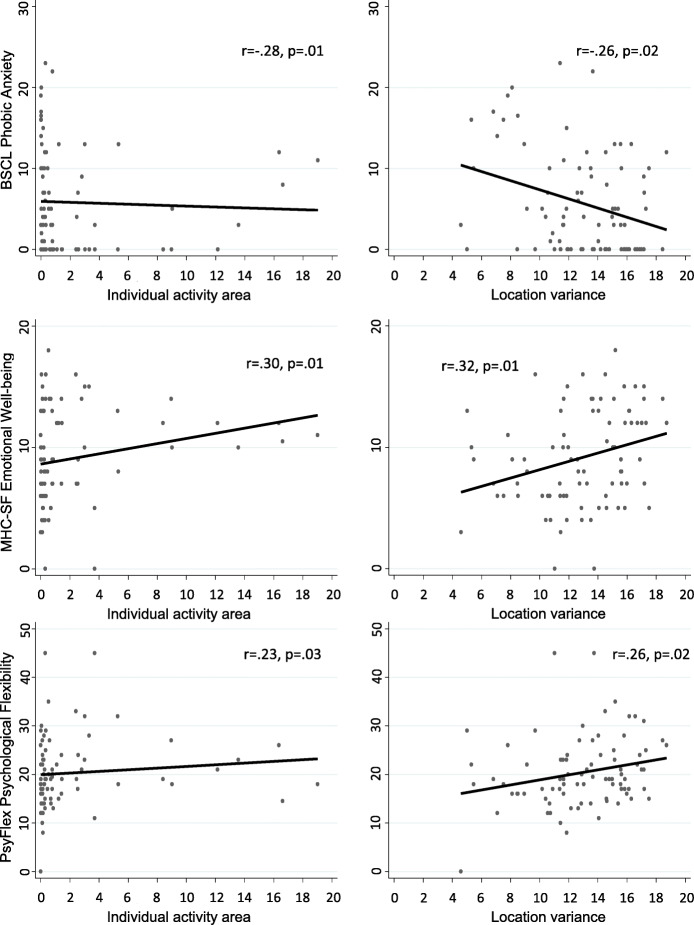
Relationship between phobic anxiety, wellbeing, and psychological flexibility & Individual activity area and Location variance.Scatterplots of the association between phobic anxiety (upper row), emotinal well-being (middle row), and psychological flexibility (bottom line) and either individual activity area (left column) or location variance (right column)

The type of symptomatology that was most closely related to spatiotemporal movement was phobic anxiety, such that higher levels of anxiety was associated with less activity area, individual activity area, location variance, and distance traveled. No other type of symptomatology was associated with more than one pattern of spatiotemporal movement. Higher levels of depression were associated with less individual activity area whereas higher levels of obsessive-compulsive symptoms, aggressiveness, and psychotic symptoms were associated with higher normalized entropy. See Table 3 and Fig. 2.

**Table 3:** Correlations of MHC-SF, BCL and Psy-Flex with estimated destinations based on the ST-DBSCAN algorithm

	Total activity area (area of convex hull area across all data points) (km^2^)		Distance travelled across the entire day (km)		Summed activity area (sum of all individual cluster areas) (km^2^)		Location variance		Entropy		Normalized entropy		Individual activity area (km^2^)		Distance travelled within destinations (km)		Length of stay within a cluster (min)	
	*r*	*p*	*r*	*p*	*r*	*p*	*r*	*p*	*r*	*p*	*r*	*p*	*r*	*p*	*r*	*p*	r	p
BSCL - Total	−0.14	0.217	−0.03	0.789	−0.17	0.132	− 0.14	0.204	0.08	0.454	0.18	0.101	−0.18	0.098	−0.12	0.265	−0.07	0.551
Somatization	−0.08	0.487	0.02	0.840	−0.09	0.415	−0.06	0.583	0.11	0.318	0.13	0.255	−0.11	0.304	−0.06	0.564	0.01	0.895
Obsessive-compulsive	−0.08	0.464	0.02	0.890	−0.11	0.304	−0.12	0.270	0.21	0.059	**0.22**	**0.050**	−0.14	0.209	−0.10	0.393	−0.18	0.107
Interpersonal sensitivity	−0.11	0.326	0.01	0.948	−0.12	0.275	−0.11	0.337	0.11	0.316	0.20	0.064	−0.14	0.213	−0.11	0.317	−0.07	0.534
Depression	−0.18	0.096	−0.09	0.402	−0.21	0.057	−0.17	0.127	0.08	0.469	0.16	0.140	**−0.22**	**0.042**	−0.18	0.113	−0.08	0.485
Anxiety	−0.07	0.534	0.04	0.729	−0.09	0.439	−0.11	0.320	0.18	0.102	0.16	0.152	−0.12	0.291	−0.10	0.370	−0.08	0.480
Hostility	−0.09	0.408	−0.04	0.693	−0.10	0.348	−0.12	0.273	0.12	0.262	**0.22**	**0.045**	−0.12	0.299	−0.13	0.256	−0.11	0.337
Phobic Anxiety	**−0.25**	**0.023**	−0.14	0.206	**−0.26**	**0.018**	**−0.26**	**0.017**	0.05	0.665	0.15	0.169	**−0.28**	**0.012**	**−0.22**	**0.044**	−0.07	0.501
Paranoid ideation	−0.10	0.378	0.02	0.844	−0.12	0.291	−0.11	0.344	0.09	0.425	0.13	0.230	−0.14	0.193	−0.08	0.446	−0.05	0.634
Psychoticism	−0.10	0.350	−0.01	0.945	−0.14	0.221	−0.13	0.253	0.09	0.419	**0.23**	**0.038**	−0.15	0.183	−0.10	0.379	−0.08	0.460
MHC-SF - Total	0.19	0.087	0.18	0.113	**0.22**	**0.044**	**0.26**	**0.019**	−0.19	0.078	−0.20	0.070	**0.24**	**0.030**	**0.29**	**0.009**	0.02	0.839
Emotional Wellbeing	**0.28**	**0.009**	**0.25**	**0.022**	**0.31**	**0.004**	**0.32**	**0.003**	−0.05	0.641	−0.20	0.067	**0.30**	**0.005**	**0.27**	**0.013**	0.04	0.710
Psychological Wellbeing	0.13	0.225	0.11	0.344	0.16	0.157	**0.22**	**0.044**	**−0.25**	**0.021**	−0.19	0.086	0.19	0.088	**0.24**	**0.031**	0.02	0.854
Social Wellbeing	0.09	0.418	0.11	0.332	0.12	0.284	0.16	0.143	−0.18	0.103	−0.15	0.179	0.13	0.242	0.21	0.055	−0.01	0.923
Psychological Flexibility	0.21	0.062	0.16	0.140	**0.24**	**0.028**	**0.26**	**0.018**	−0.15	0.187	−0.15	0.167	**0.23**	**0.034**	0.21	0.052	0.16	0.150

*Note:* ST-DBSCAN parameter setting: *eps1* = 200 m, *eps2* = 20 min and *minpts* = 10; *r* = Spearman rank correlation coefficient; *p* = *p*-value

### Relation between spatiotemporal movement and wellbeing and psychological flexibility (hypothesis 3)

In contrast to symptoms, wellbeing and psychological flexibility were consistently associated with patients’ spatiotemporal movement patterns. In particular higher levels of emotional wellbeing and to a lesser degree psychological wellbeing were associated to most spatiotemporal patterns. Social wellbeing was not associated with any spatiotemporal pattern. Finally, the more psychologically flexible patients were, the greater their summed activity area, location variance, and individual activity area. See Table 3 and Fig. 2.

## Discussion

This study is the first to examine the spatiotemporal characteristics of a large group of patients treated in both inpatient and outpatient contexts. Results showed that an algorithm can differentiate logical patterns in the spatiotemporal movement of both patient groups. Symptomatology, with the exception of agoraphobic anxiety, was not consistently related to spatiotemporal movements. In contrast, measures of wellbeing and psychological flexibility were consistently related, with variability of movement emerging as the most consistent relation. This study provides a benchmark for spatiotemporal characteristics of patients presenting for treatment, which can serve as a possible marker for patient functioning.

The differences we documented in spatiotemporal movement patterns for inpatients and outpatients were logical. For example, compared to inpatients, outpatients had a greater number of destinations at home, other locations, and fewer destinations at the hospital. In sum, these results demonstrate both the feasibility and proof of method for using GPS as a passive, non-intrusive assessment of spatiotemporal movement when patients present for treatment.

Results also showed that higher depressive symptoms were related to small activity area. These results were consistent with a-priori predictions and lend support to the validity of using GPS to measure spatiotemporal movement. In addition, normalized entropy, a measure of variability, was not related to symptoms in general. This was consistent with predictions. Given the paucity of GPS-based research in patients diagnosed with mental disorders, care should be taken when interpreting the other significant relations.

In contrast to symptomatology, patient’s movement patterns are most consistently associated with wellbeing, in particular emotional wellbeing (medium effect size). This is consistent with our prediction and with studies that have reported an association between overall purposeful physical activity and wellbeing [19]. Also, partially consistent with our predictions was the observed association between spatiotemporal movement and psychological flexibility (medium effect size). Given that psychological flexibility describes the ability to engage in valued activities despite symptoms [20], it suggests a meaningful intervention target and assessment outcome.

The clinical implications of this study involve utilizing a data source that is readily produced by any patient with a smartphone. As research in this area progresses, it is conceivable that the meta-information of spatiotemporal clustering can be harnessed to help clinicians assess patterns and changes in patterns during treatment. The potential of this approach lies in the ease of data collection. These potential advantages must be carefully balanced against concerns of ethics and data security [21], and these issues need to be considered fully and collaboratively by researchers, clinicians, and patients.

This study needs to be considered in light of several limitations. First, participants used study-issued smartphones, not their personal ones. Whereas this allowed us to maximize their data security and protect their privacy, we are unable to know whether this affected the degree to which they carried the phone. Second, we were unable to validate that participants carried the phone with them wherever they went. Third, a patient was included into the study if at least 1000 signals were available on a particular day, which corresponds to 1.4 h of signals. The number of destinations and time within a destination may be underestimated if the signal was not recorded all day long. However, it is currently unknown what accounted for missing data (turning off the phone, poor cell reception, etc.) and whether and in what way this impacts the results. Future studies should focus on the reasons for missing GPS recordings.

These limitations notwithstanding, this is the first study to document the feasibility of collecting GPS data from a large group of routine patients treated both in inpatient and outpatient settings. The study also documented the robustness of these spatiotemporal algorithms as evidenced by the resulting logical differential patterns observed between inpatients and outpatients. Other algorithms might lead to different results and should be tested. This study contributes to literatures on mental health, digital health, and digital phenotyping [22–24]. The results point to the fact that patterns of movement (e.g., distance, number of destinations, variability of destinations, etc.) may serve as a marker of functioning and wellbeing. By establishing the parameter of spatiotemporal movement and testing across diagnostic groups and treatment settings, this study and others like it, are consistent with current initiatives to identify processes relevant across diagnostic boundaries of mental illness and health (research domain criteria (RDoC)) [25]. Given the importance of physical movement on mental health [26], and for general health [1], more work in this area is warranted. Future studies should link the spatiotemporal parameters with finer grained assessments of patients’ state levels of symptomatology, wellbeing, and other psychological processes that mediate their impact.

## Supplementary Information

**Additional file 1 Table S1.** Sensitivity analysis corresponding to Table 2 in the main article: Number of destinations and number of destinations per day estimated by the parameter setting *eps1* = 200 m, *eps2* = 20 min and *minpts* = 10 of the ST-DBSCAN algorithm – based on a restricted dataset including only patients for whom more than 60% of all daily GPS signals were recorded.

**Additional file 2 Table S2.** Characteristics of the estimated destinations by the parameter set *eps1* = 200 m, *eps2* = 20 min and *minpts* = 10.

## Data Availability

The raw data will not be publicly available because it contains information that could compromise the participant’s privacy. Aggregated data that does not compromise privacy will be available from the author upon reasonable request.

## Abbreviations

GPS: Global Positioning System
ST-DBSCAN: Spatial Temporal Density-Based Spatial Clustering of Applications with Noise
ESM: Event Sampling Methodology

## References

[1] Nathan R, Getz WM, Revilla E, Holyoak M, Kadmon R, Saltz D (2008). A movement ecology paradigm for unifying organismal movement research. Proceed Nat Acad Sci USA.

[2] Bize R, Johnson JA, Plotnikoff RC (2007). Physical activity level and health-related quality of life in the general adult population: A systematic review. Preventive Med.

[3] Mammen G, Faulkner G (2013). Physical activity and the prevention of depression: a systematic review of prospective studies. Am J Prev Med.

[4] Vallée J, Cadot E, Roustit C, Parizot I, Chauvin P (2011). The role of daily mobility in mental health inequalities: the interactive influence of activity space and neighbourhood of residence on depression. Soc Sci Med.

[5] American Psychiatric Association (2013). Anxiety Disorders. Diagnostic and Statistical Manual of Mental Disorders 5th ed. Washington D.C.

[6] American Psychiatric Association (2013). Somatic Symptom and Related Disorders. Diagnostic and Statistical Manual of Mental Disorders 5th ed. Washington D.C.

[7] Lamers SMA, Westerhof GJ, Bohlmeijer ET, Ten Klooster PM, Keyes CLM (2011). Evaluating the psychometric properties of the mental health continuum-short form (MHC-SF). J Clin Psychol.

[8] Gloster AT, Klotsche J, Ciarrochi J, Eifert G, Sonntag R, Wittchen HU (2017). Increasing valued behaviors precedes reduction in suffering: findings from a randomized controlled trial using ACT. Behav Res Ther.

[9] Gloster AT, Meyer AH, Lieb R (2017). Psychological flexibility as a malleable public health target: evidence from a representative sample. J Context Behav Sci..

[10] Gloster AT, Gerlach AL, Hamm A, Höfler M, Alpers GW, Kircher T (2015). 5HTT is associated with the phenotype psychological flexibility: results from a randomized clinical trial. Eur Arch Psychiatry Clin Neurosci.

[11] Saeb S, Zhang M, Karr CJ, Schueller SM, Corden ME, Kording KP (2015). Mobile phone sensor correlates of depressive symptom severity in daily-life behavior: an exploratory study. J Med Internet Res.

[12] Brusilovskiy E, Townley G, Snethen G, Salzer MS (2016). Social media use, community participation and psychological well-being among individuals with serious mental illnesses. Comput Human Behav.

[13] Villanueva J, Meyer AH, Rinner MTB, Firsching VJ, Benoy C, Brogli S, et al. “Choose change”: design and methods of an acceptance and commitment therapy effectiveness trial for transdiagnostic treatment-resistant patients. BMC Psychiatry. 2019;19(1):173. 10.1186/s12888-019-2109-4. 10.1186/s12888-019-2109-4 PMC6558686 31182051

[14] Hoang HT, Pham QV, Hwang WJ (2020). Spatial-temporal-dbscan-based user clustering and power allocation for sum rate maximization in millimeter-wave noma systems. Symmetry.

[15] Derogatis LR (1983). The brief symptom inventory: an introductory report. Psychol Med.

[16] Benoy C, Knitter B, Schumann I, Bader K, Walter M, Gloster AT (2019). Treatment sensitivity: its importance in the measurement of psychological flexibility. J Context Behav Sci.

[17] Birant D, Kut A (2007). ST-DBSCAN: an algorithm for clustering spatial-temporal data. Data Knowl Eng.

[18] R Core Team (2018). R: a language and environment for statistical computing.

[19] SJH B, Asare M (2011). Physical activity and mental health in children and adolescents: A review of reviews. Br J Sports Med. British Association of Sport and Excercise Medicine.

[20] Kashdan TB, Rottenberg J (2010). Psychological flexibility as a fundamental aspect of health. Clin Psychol Rev.

[21] Martinez-Martin N, Insel TR, Dagum P, Greely HT, Cho MK. Data mining for health: staking out the ethical territory of digital phenotyping. npj Digital Med 1, 68. 2018;1(1):1–5. 10.1038/s41746-018-0075-8. 10.1038/s41746-018-0075-8 PMC6550156 31211249

[22] Insel TR (2018). Digital phenotyping: a global tool for psychiatry. World Psychiatry.

[23] Aledavood T, Triana Hoyos AM, Alakörkkö T, Kaski K, Saramäki J, Isometsä E (2017). Data collection for mental health studies through digital platforms: requirements and Design of a Prototype. JMIR Res Protoc.

[24] Kirchner TR, Shiffman S (2016). Spatio-temporal determinants of mental health and well-being: advances in geographically-explicit ecological momentary assessment (GEMA). Social Psychiatry and Psychiatric Epidemiology.

[25] Insel T, Cuthbert B, Garvey M, Heinssen R, Pine DS, Quinn K (2010). Research Domain Criteria (RDoC): Toward a new classification framework for research on mental disorders. American Journal of Psychiatry. American Psychiatric Association.

[26] Letsinger AC, Granados JZ, Little SE, Lightfoot JT (2019). Alleles associated with physical activity levels are estimated to be older than anatomically modern humans. Calafell F, editor. PLoS One.

