# Supplementary material for: The spatiotemporal movement of patients in and out of a psychiatric hospital: an observational GPS study
Source: BMC Psychiatry. 2021 Mar 24;21:165. doi: 10.1186/s12888-021-03147-9 (PMC7992323; doi:10.1186/s12888-021-03147-9)
Supplement: Supplementary file 1 — Additional file 1 Table S1. Sensitivity analysis corresponding to Table 2 in the main article: Number of destinations and number of destinations per day estimated by the parameter setting eps1 = 200 m, eps2 = 20 min and minpts = 10 of the ST-DBSCAN algorithm – based on a restricted dataset including only patients for whom more than 60% of all daily GPS signals were recorded. [file 12888_2021_3147_MOESM1_ESM.docx]

**Additional File 1**

**Table.** Sensitivity analysis corresponding to Table 2 in the main article: Number of destinations and number of destinations per day estimated by the parameter setting *eps1* = 200 meter, *eps2* = 20 minutes and *minpts* = 10 of the ST-DBSCAN algorithm – based on a restricted dataset including only patients for whom more than 60% of all daily GPS signals were recorded.

|  |  |  |  |  |
| --- | --- | --- | --- | --- |
|  | Inpatients | Outpatients | *p* value | All |
|  | (n=37) | (n=28) | (inpatients versus outpatients) | (n=65) |
|  |  |  |  |  |
| Total number of destinations | 712 (100.0%) | 1421 (100.0%) |  | 2133 (100.0%) |
| at home | 60 (8.4%) | 302 (21.3%) | <0.001 | 362 (17.0%) |
| at hospital | 268 (37.6%) | 8 (0.6%) |  | 276 (12.9%) |
| at other location | 325 (45.6%) | 813 (57.2%) |  | 1138 (53.4%) |
| in transit | 54 (7.6%) | 122 (8.6%) |  | 176 (8.3%) |
| location unclear^1^ | 5 (0.7%) | 176 (12.4%) |  | 181 (8.5%) |
|  |  |  |  |  |
| Average number of destinations | 10.8 (5.7) | 9.5 (6.2) |  | 9.9 (6.0) |
| per patient and day^2^ | 9.5 (7 - 13) | 9 (5 - 13) | 0.116 | 9 (6 - 13) |
| at home^2^ | 0.9 (1.7) | 2.0 (2.0) |  | 1.7 (2.0) |
|  | 0 (0 - 1) | 2 (0 - 3) | <0.001 | 1 (0 - 3) |
| at hospital^2^ | 4.1 (3.2) | 0.1 (0.3) |  | 1.3 (2.6) |
|  | 3 (1 - 6) | 0 (0 - 0) | <0.001 | 0 (0 - 1) |
| at other location^2^ | 4.9 (5.8) | 5.5 (6.0) |  | 5.3 (5.9) |
|  | 2 (0 - 8) | 4 (0 - 9) | 0.642 | 4 (0 - 8) |
| in transit^2^ | 0.8 (0.4) | 0.8 (0.4) |  | 0.8 (0.4) |
|  | 1 (1 - 1) | 1 (1 - 1) | 0.954 | 1 (1 - 1) |
| location unclear^1,2^ | 0.1 (0.4) | 1.2 (3.3) |  | 0.8 (2.8) |
|  | 0 (0 - 0) | 0 (0 - 0) | 0.006 | 0 (0 - 0) |
| Summed activity area (sum of all individual cluster areas) (km^2^) | 34.3 (118.6) | 138.8 (571.5) |  | 106.7 (482.2) |
|  | 1.4 (0.1 - 3.7) | 2.4 (0.3 - 18.3) | 0.187 | 2.2 (0.2 - 11.3) |
|  |  |  |  |  |
| Percent time within a cluster in repect to the recorded total time |  |  |  |  |
| at home^2^ | 9.5 (20.2) | 31.7 (30.4) |  | 19.1 (27.3) |
|  | 0.0 (0.0 - 8.1) | 30.3 (0.0 - 53.4) | 0.001 | 0.0 (0.0 - 34.3) |
| at Hospital^2^ | 37.8 (26.9) | 0.6 (2.3) |  | 21.8 (27.4) |
|  | 32.2 (19.0 - 53.3) | 0.0 (0.0 - 0.0) | <0.001 | 6.8 (0.0 - 34.5) |
| at other location^2^ | 18.2 (19.3) | 22.8 (22.8) |  | 20.2 (20.8) |
|  | 15.1 (0.0 - 24.9) | 14.9 (6.2 - 38.4) | 0.488 | 15.1 (1.3 - 31.8) |
| in transit^2^ | 34.2 (26.0) | 30.7 (18.9) |  | 32.7 (23.1) |
|  | 36.6 (6.4 - 56.9) | 30.8 (17.1 - 43.1) | 0.615 | 32.6 (15.3 - 48.7) |
| location unclear^1,2^ | 0.2 (0.9) | 14.2 (31.2) |  | 6.3 (21.5) |
|  | 0.0 (0.0 - 0.0) | 0.0 (0.0 - 0.0) | 0.178 | 0.0 (0.0 - 0.0) |
|  |  |  |  |  |
| ^1^ Locations unclear since home address not known and cluster status therefore not unequivocally attributable. | | | | |
| ^2^ Statistics denote mean (SD) at first row, and median (25. – 75. percentile) at second row. | | | | |
