# Supplementary material for: The spatiotemporal movement of patients in and out of a psychiatric hospital: an observational GPS study
Source: BMC Psychiatry. 2021 Mar 24;21:165. doi: 10.1186/s12888-021-03147-9 (PMC7992323; doi:10.1186/s12888-021-03147-9)
Supplement: Supplementary file 2 — Additional file 2 Table S2. Characteristics of the estimated destinations by the parameter set eps1 = 200 m, eps2 = 20 min and minpts = 10. [file 12888_2021_3147_MOESM2_ESM.docx]

**Additional File 2**

**Table.** Characteristics of the estimated destinations by the parameter set *eps1* = 200 meter, *eps2* = 20 minutes and *minpts* = 10

|  |  |  |  |  |  |  |  |  |
| --- | --- | --- | --- | --- | --- | --- | --- | --- |
|  |  | Inpatients | | Outpatients | |  | All | |
|  |  | n=53 | | n=31 | |  | n=84 | |
|  |  | Number of clusters | Mean (SD) | Number of clusters | Mean (SD) | *p* value (inpatients versus outpatients) | Number of clusters | Mean (SD) |
|  |  |  | Median (p25 – p75) |  | Median (p25 – p75) |  |  | Median (p25 – p75) |
| Total activity area (area of convex hull area across all data points) (km^2^) |  | 2041 | 21.1 (94.8) | 1728 | 110.7 (501.8) |  | 3769 | 61.1 (345.0) |
|  |  |  | 0.32 (0.04 - 2.67) |  | 2.95 (0.45 - 13.55) | <0.001 |  | 1.08 (0.05 - 6.01) |
| Summed activity area (sum of all individual cluster areas) (km^2^) |  | 2041 | 15.1 (83.9) | 1728 | 106.4 (496.3) |  | 3769 | 55.8 (340.0) |
|  |  |  | 0.30 (0.04 - 2.36) |  | 2.12 (0.25 - 10.14) | <0.001 |  | 0.69 (0.06 - 3.72) |
| Individual activity area (km²) |  |  |  |  |  |  |  |  |
|  | at home^2^ | 139 | 0.02 (0.05) | 345 | 0.02 (0.05) |  | 484 | 0.02 (0.05) |
|  |  |  | 0.00 (0.00 - 0.01) |  | 0.00 (0.00 - 0.02) | 0.326 |  | 0.00 (0.00 - 0.01) |
|  | at Hospital^2^ | 932 | 0.01 (0.04) | 14 | 0.03 (0.03) |  | 946 | 0.01 (0.04) |
|  |  |  | 0.00 (0.00 - 0.01) |  | 0.02 (0.00 - 0.05) | 0.750 |  | 0.00 (0.00 - 0.01) |
|  | at other location^2^ | 726 | 0.27 (0.70) | 996 | 0.13 (0.45) |  | 1722 | 0.19 (0.58) |
|  |  |  | 0.05 (0.01 - 0.19) |  | 0.04 (0.01 - 0.10) | 0.407 |  | 0.04 (0.01 - 0.13) |
|  | in transit^2^ | 135 | 26.02 (112.44) | 152 | 138.95 (565.48) |  | 287 | 85.83 (421.83) |
|  |  |  | 0.00 (0.00 - 1.20) |  | 2.20 (0.13 - 24.50) | <0.001 |  | 0.41 (0.00 - 8.83) |
|  | location unclear^1,2^ | 109 | 0.08 (0.19) | 221 | 0.07 (0.24) |  | 330 | 0.07 (0.22) |
|  |  |  | 0.00 (0.00 - 0.07) |  | 0.02 (0.00 - 0.05) | 0.845 |  | 0.01 (0.00 - 0.06) |
|  |  | Inpatients | | Outpatients | |  | All | |
|  |  | n=53 | | n=31 | |  | n=84 | |
|  |  | Number of clusters | Mean (SD) | Number of clusters | Mean (SD) | *p* value (inpatients versus outpatients) | Number of clusters | Mean (SD) |
|  |  |  | Median (p25 – p75) |  | Median (p25 – p75) |  |  | Median (p25 – p75) |
| Distance travelled across the entire day (km) |  | 2041 | 19.4 (27.1) | 1728 | 35.5 (94.2) |  | 3769 | 26.6 (66.5) |
|  |  |  | 11.27 (6.98 - 19.17) |  | 13.56 (4.95 - 28.75) | 0.434 |  | 11.90 (6.49 - 24.16) |
| Location variance |  | 2041 | 11.4 (4.3) | 1728 | 13.8 (4.7) |  | 3769 | 12.5 (4.7) |
|  |  |  | 11.84 (7.72 - 14.24) |  | 14.81 (13.04 - 16.34) | <0.001 |  | 13.44 (8.67 - 15.66) |
| Entropy |  | 2041 | 1.4 (0.4) | 1728 | 1.2 (0.5) |  | 3769 | 1.3 (0.5) |
|  |  |  | 1.39 (1.08 - 1.70) |  | 1.32 (0.88 - 1.61) | 0.007 |  | 1.34 (1.01 - 1.65) |
| Normalized entropy |  | 2041 | 0.6 (0.2) | 1728 | 0.5 (0.2) |  | 3769 | 0.6 (0.2) |
|  |  |  | 0.61 (0.46 - 0.72) |  | 0.51 (0.40 - 0.61) | <0.001 |  | 0.55 (0.43 - 0.68) |
| Distance travelled within destinations (km) |  |  |  |  |  |  |  |  |
|  | at home^2^ | 139 | 1.45 (1.99) | 345 | 0.72 (1.03) |  | 484 | 0.93 (1.42) |
|  |  |  | 0.68 (0.11 - 2.02) |  | 0.37 (0.03 - 0.96) | <0.001 |  | 0.43 (0.05 - 1.14) |
|  | at Hospital^2^ | 932 | 1.28 (1.76) | 14 | 0.66 (0.62) |  | 946 | 1.27 (1.75) |
|  |  |  | 0.62 (0.16 - 1.75) |  | 0.71 (0.02 - 1.09) | 0.169 |  | 0.62 (0.16 - 1.73) |
|  | at other location^2^ | 726 | 1.91 (2.35) | 996 | 1.34 (1.57) |  | 1722 | 1.58 (1.96) |
|  |  |  | 1.06 (0.54 - 2.36) |  | 0.81 (0.53 - 1.54) | <0.001 |  | 0.89 (0.53 - 1.85) |
|  | in transit^2^ | 135 | 9.20 (22.36) | 152 | 27.89 (60.01) |  | 287 | 19.10 (47.15) |
|  |  |  | 0.29 (0.03 - 6.74) |  | 8.89 (1.99 - 25.88) | <0.001 |  | 3.23 (0.13 - 16.82) |
|  | location unclear^1,2^ | 109 | 1.22 (1.57) | 221 | 1.07 (2.09) |  | 330 | 1.12 (1.93) |
|  |  |  | 0.62 (0.19 - 1.72) |  | 0.66 (0.25 - 1.19) | 0.488 |  | 0.64 (0.19 - 1.32) |
| Length of stay within a cluster (min) |  |  |  |  |  |  |  |  |
|  | at home^2^ | 139 | 111.49 (154.11) | 345 | 241.56 (279.15) |  | 484 | 204.21 (256.43) |
|  |  |  | 49.50 (8.75 - 130.40) |  | 129.53 (46.68 - 357.55) | <0.001 |  | 99.68 (28.74 - 300.71) |
|  | at Hospital^2^ | 932 | 79.50 (107.26) | 14 | 152.25 (243.54) |  | 946 | 80.57 (110.58) |
|  |  |  | 38.16 (9.41 - 105.23) |  | 78.55 (7.95 - 132.90) | 0.621 |  | 38.29 (9.40 - 105.38) |
|  |  | Inpatients | | Outpatients | |  | All | |
|  |  | n=53 | | n=31 | |  | n=84 | |
|  |  | Number of clusters | Mean (SD) | Number of clusters | Mean (SD) | *p* value (inpatients versus outpatients) | Number of clusters | Mean (SD) |
|  |  |  | Median (p25 – p75) |  | Median (p25 – p75) |  |  | Median (p25 – p75) |
|  | at other location^2^ | 726 | 52.13 (88.76) | 996 | 51.50 (123.35) |  | 1722 | 51.76 (110.07) |
|  |  |  | 15.21 (3.60 - 66.77) |  | 7.51 (2.31 - 41.23) | <0.001 |  | 10.28 (2.60 - 54.52) |
|  | in transit^2^ | 135 | 229.68 (288.34) | 152 | 404.25 (339.58) |  | 287 | 323.11 (328.06) |
|  |  |  | 80.28 (0.54 - 393.60) |  | 350.77 (95.74 - 642.98) | <0.001 |  | 235.02 (15.15 - 560.59) |
|  | location unclear^1,2^ | 109 | 44.36 (69.51) | 221 | 162.95 (276.65) |  | 330 | 123.78 (236.40) |
|  |  |  | 13.35 (3.68 - 58.03) |  | 31.12 (5.63 - 228.83) | <0.001 |  | 25.20 (4.75 - 111.22) |
| Percent time within a cluster in repect to the recorded total time |  |  |  |  |  |  |  |  |
|  | at home^2^ | 139 | 7.3 (14.4) | 345 | 29.6 (31.0) |  | 484 | 15.5 (24.4) |
|  |  |  | 0.0 (0.0 - 6.6) |  | 31.2 (0.0 - 57.8) | <0.001 |  | 0.0 (0.0 - 30.4) |
|  | at Hospital^2^ | 932 | 45.9 (28.2) | 14 | 0.6 (2.1) |  | 946 | 29.2 (31.4) |
|  |  |  | 43.1 (22.1 - 71.2) |  | 0.0 (0.0 - 0.0) | <0.001 |  | 16.5 (0.0 - 56.5) |
|  | at other location^2^ | 726 | 22.3 (20.0) | 996 | 22.1 (23.8) |  | 1722 | 22.2 (21.3) |
|  |  |  | 18.3 (3.7 - 33.6) |  | 13.0 (0.0 - 43.5) | 0.586 |  | 17.6 (3.2 - 37.0) |
|  | in transit^2^ | 135 | 20.0 (18.6) | 152 | 26.6 (16.9) |  | 287 | 22.4 (18.2) |
|  |  |  | 16.1 (2.7 - 30.6) |  | 25.7 (14.3 - 41.3) | 0.058 |  | 20.2 (5.1 - 32.6) |
|  | location unclear^1,2^ | 109 | 4.6 (16.1) | 221 | 21.1 (37.0) |  | 330 | 10.7 (26.9) |
|  |  |  | 0.0 (0.0 - 0.0) |  | 0.0 (0.0 - 55.1) | 0.084 |  | 0.0 (0.0 - 0.0) |
|  |  |  |  |  |  |  |  |  |
| Percent of clusters of all clusters within a day |  |  |  |  |  |  |  |  |
|  | at home^2^ | 139 | 6.3 (11.8) | 345 | 19.0 (20.7) |  | 484 | 11.0 (16.7) |
|  |  |  | 0.0 (0.0 - 8.7) |  | 15.5 (0.0 - 28.6) | 0.002 |  | 0.0 (0.0 - 18.9) |
|  | at Hospital^2^ | 932 | 48.6 (27.8) | 14 | 0.7 (1.4) |  | 946 | 31.0 (32.1) |
|  |  |  | 48.5 (22.2 - 72.4) |  | 0.0 (0.0 - 1.3) | <0.001 |  | 20.5 (0.0 - 56.8) |
|  |  | Inpatients | | Outpatients | |  | All | |
|  |  | n=53 | | n=31 | |  | n=84 | |
|  |  | Number of clusters | Mean (SD) | Number of clusters | Mean (SD) | *p* value (inpatients versus outpatients) | Number of clusters | Mean (SD) |
|  |  |  | Median (p25 – p75) |  | Median (p25 – p75) |  |  | Median (p25 – p75) |
|  | at other location^2^ | 726 | 33.2 (25.1) | 996 | 47.5 (34.2) |  | 1722 | 38.5 (29.4) |
|  |  |  | 33.3 (14.8 - 50.0) |  | 60.0 (0.0 - 74.0) | 0.048 |  | 36.7 (13.3 - 63.6) |
|  | in transit^2^ | 135 | 7.0 (4.2) | 152 | 10.0 (3.3) |  | 287 | 8.1 (4.2) |
|  |  |  | 6.5 (4.5 - 9.1) |  | 9.1 (8.0 - 11.3) | <0.001 |  | 8.0 (5.7 - 9.8) |
|  | location unclear^1,2^ | 109 | 4.9 (16.3) | 221 | 22.9 (39.5) |  | 330 | 11.5 (28.4) |
|  |  |  | 0.0 (0.0 - 0.0) |  | 0.0 (0.0 - 80.0) | 0.086 |  | 0.0 (0.0 - 0.0) |
|  |  |  |  |  |  |  |  |  |
| ^1^ Locations unclear since home address not known and cluster status therefore not unequivocally attributable. | | | | | | | | |
| ^2^ Statistics denote mean (SD) and median (25. – 75. percentile) | | | | | | | | |
